# Diabetic Nephropathy and CKD—Analysis of Individual Patient Serum Creatinine Trajectories: A Forgotten Diagnostic Methodology for Diabetic CKD Prognostication and Prediction

**DOI:** 10.3390/jcm4071348

**Published:** 2015-06-26

**Authors:** Macaulay Amechi Chukwukadibia Onuigbo, Nneoma Agbasi

**Affiliations:** 1 Medicine, Mayo Clinic College of Medicine, Rochester, MN 55905, USA; 2 North East London National Health Service Foundation Trust, Barley Lane, Ilford, Essex, 1G3 8XJ, UK; E-Mail: nnoms@aol.com; 3 Department of Nephrology, Mayo Clinic Health System, MCHS Eau Claire, 1221 Whipple Street, Eau Claire, WI 54702, USA

**Keywords:** acute kidney injury (AKI), chronic kidney disease (CKD), creatinine, end stage renal disease (ESRD), national kidney foundation kidney disease outcomes quality initiative (NKF KDOQI), renoprevention, serum creatinine trajectory, syndrome of late onset renal failure from angiotensin blockade (LORFFAB), syndrome of rapid onset end stage renal disease (SORO-ESRD)

## Abstract

Creatinine is produced in muscle metabolism as the end-product of creatine phosphate and is subsequently excreted principally by way of the kidneys, predominantly by glomerular filtration. Blood creatinine assays constitute the most common clinically relevant measure of renal function. The use of individual patient-level real-time serum creatinine trajectories provides a very attractive and tantalizing methodology in nephrology practice. Topics covered in this review include acute kidney injury (AKI) with its multifarious rainbow spectrum of renal outcomes; the stimulating vicissitudes of the diverse patterns of chronic kidney disease (CKD) to end-stage renal disease (ESRD) progression, including the syndrome of rapid onset end stage renal disease (SORO-ESRD); the syndrome of late onset renal failure from angiotensin blockade (LORFFAB); and post-operative AKI linked with the role of intra-operative hypotension in patients with diabetes mellitus and suspected diabetic nephropathy with CKD. We conclude that the study of individual patient-level serum creatinine trajectories, albeit a neglected and forgotten diagnostic methodology for diabetic CKD prognostication and prediction, is a most useful diagnostic tool, both in the short-term and in the long-term practice of nephrology. The analysis of serum creatinine trajectories, both in real time and retrospectively, indeed provides supplementary superior diagnostic and prognostic insights in the management of the nephrology patient.

## 1. Introduction

### 1.1. Diabetic Nephropathy

Diabetic nephropathy or diabetic kidney disease is defined by characteristic structural and functional changes, with the predominant structural changes described being mesangial expansion, glomerular basement membrane thickening, and glomerular sclerosis. Functional characteristics include hyperfiltration, microalbuminuria, macroalbuminuria with incipient progressive proteinuria that is then often followed by a slowly progressive decline in glomerular filtration rate (GFR) and, over time, ending inexorably in symptomatic end-stage renal disease (ESRD) requiring renal replacement therapy [1]. For unclear reasons, the degree of albuminuria is not necessarily linked to disease progression in patients with diabetic nephropathy associated with either type 1 or type 2 diabetes [2,3,4]. In a 2010 Joslin Kidney Study report of 79 patients with type 1 diabetes who followed for a mean of 12 years after the onset of moderately increased albuminuria, 23 patients progressed to advanced disease (GFR less than 60 mL/min), 11 of whom had either stable, moderately increased albuminuria or regression to normal albuminuria [2]. The other 12 patients developed proteinuria that usually accompanied, but did not precede, the decline in GFR [2]. It must therefore be recognized that diabetic nephropathy is a very heterogenous disease entity [5]. Tsalamandris *et al.* had similar observations when, of 40 patients with diabetes, 15 developed progressive increase in albuminuria without decline in GFR, 13 had progressive increase in albuminuria concurrent with decreasing GFR, and 12 (eight type 2 diabetics and four type 1 diabetics) exhibited decreasing GFR values albeit without significant increase in albuminuria [6].

From the foregoing, and as was the case in this presentation, adult diabetic patients with CKD seen at the Renal Unit of the Mayo Clinic Health System in Northwestern Wisconsin, with or without documented albuminuria or proteinuria, are all therefore assumed to have some form of diabetic nephropathy [1,2,3,4,5,6].

### 1.2. Creatinine-Metabolism, Chemical Structure, and Excretion in the Urine

Creatinine is produced in muscle metabolism as the end product of creatine phosphate and is subsequently excreted principally by way of the kidneys, predominantly by glomerular filtration [7]. Blood assays for creatinine constitute the most commonly used measure of the presence and progression of CKD. Thus, the level of the serum creatinine in a subject is a general reflection of the level of kidney function. With kidney disease and loss of nephrons, the level of serum creatinine would therefore show an upward trend. Conversely, with improving kidney function, such as that seen following acute kidney injury (AKI), the level of serum creatinine will then trend downwards. Accordingly, serum creatinine is the most widely used assay to measure the presence and progression of CKD [8]. The use of individual patient-level real-time monitoring of serum creatinine translations, the so-called serum creatinine trajectories, provides a very attractive and tantalizing methodology in general nephrology practice. We have investigated and reviewed the utility and place of serum creatinine trajectories in patient care, covering both inpatient hospital care, including critical care medicine, as well as long-term outpatient CKD patient care. We have covered pertinent topics such as AKI with its multifarious rainbow spectrum of renal outcomes; the stimulating vicissitudes of the diverse patterns of CKD to ESRD progression, including our latest overview of the syndrome of rapid onset end-stage renal disease (SORO-ESRD); the syndrome of late onset renal failure from angiotensin blockade (LORFFAB); and post-operative AKI from intra-operative hypotension in patients with diabetes mellitus and suspected diabetic nephropathy with CKD. These patients were all representative cases managed in the Renal Unit of the Mayo Clinic Health System, Eau Claire, Northwestern Wisconsin, USA.

## 2. Main Review

### 2.1. Serum Creatinine Trajectories in Diabetic Nephropathy

#### i. Acute Kidney Injury (AKI): the Rainbow Spectrum of Renal Outcomes in AKI in CKD Patients

The general assumption among physicians and some nephrologists is that the impact of AKI on renal function is usually short-lived and transient, with the typical course of an expected recovery of renal function in most instances [9,10,11,12]. Nevertheless, growing mutually anecdotal and objective evidence in the nephrology literature is contrarian, and demonstrates that, quite often, much less renal recovery occurs after AKI on CKD events, and this is generally unrecognized by physicians [12]. Undeniably, there is new and cumulative evidence in the AKI literature that AKI not only propagates CKD, but that AKI directly leads to acute yet irreversible ESRD and the need for permanent renal replacement therapy, the so-called newly described syndrome of rapid onset end stage renal disease [13].

We will now report cases of AKI in diabetic patients, demonstrating a spectrum of differing renal outcomes, accompanied by graphs of serum creatinine trajectories [14].

##### a. Rapid and Full Recovery of Renal Function Following AKI on CKD

**Figure 1:**
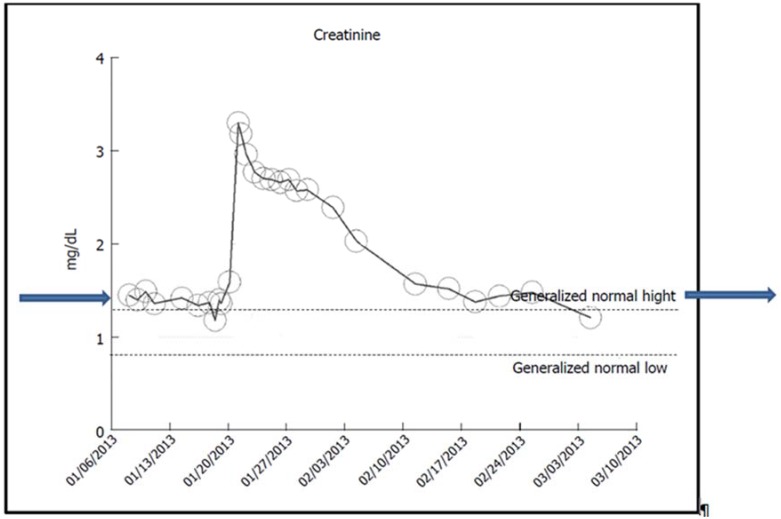
Serum creatinine trajectory in an obese hypertensive with glucose intolerance showing rapid and full recovery of renal function following AKI on CKD from MRSA bacteremia.

A 48-year-old obese Caucasian male patient with nearly 10 years of hypertension, glucose intolerance, A1c of 6%, and stage II CKD (baseline serum creatinine of 1.2 mg/dL) developed AKI on CKD in January 2013 from methicillin-resistant *Staphylococcus aureus* (MRSA) bacteremia [12]. Urine albumin creatinine ratio (UACR) was not available, 24-h urinary protein excretion was normal at 25 mg, and renal ultrasound was normal. Concurrent ACE inhibition (Benazepril) was discontinued, whereas Amlodipine was continued for hypertension control. He received multiple courses of different parenteral antibiotics including Nafcillin, Vancomycin, and Cubicin, as indicated by susceptibility test results. A kidney biopsy revealed acute interstitial nephritis. Serum creatinine peaked at 3.3 mg/dL (Figure 1). Renal function improved with conservative therapy alone. A month later, his kidney function was fully back to normal again (Figure 1). The cause of renal failure was MRSA septicemia.

##### b. The Syndrome of Rapid Onset ESRD (SORO-ESRD) Following AKI on CKD in a Diabetic Patient

**Figure 2:**
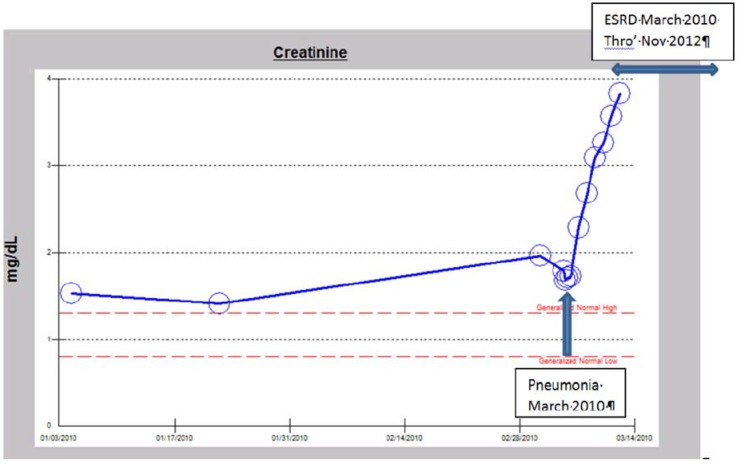
Serum creatinine trajectory demonstrating rapid-onset, acute yet irreversible ESRD requiring RRT following pneumonia in an obese, hypertensive, diabetic male Caucasian with III CKD in March 2010.

A 72-year-old morbidly obese Caucasian male patient with type II diabetes mellitus since 2005 (on insulin and metformin), hypertension since 2005, III CKD, atrial fibrillation, promyelocytic leukemia in remission, and steroid-dependent severe chronic obstructive pulmonary disease (COPD) was admitted with worsening dyspnea and edema in early March 2010. His baseline serum creatinine was 1.9 mg/dL. His A1c was 6%, dipstick urinalysis was 1+ proteinuria, UACR was 41 mg/g (0–30), and 24-h protein excretion was 91 mg (42–225). Renal ultrasound was not available. An ophthalmology evaluation in March 2012 revealed no diabetic retinopathy. The work-up confirmed a left lower lobe pneumonia and he received intravenous antibiotics including Levofloxacin, high-dose steroids, and Nebulizer treatments. Metformin was discontinued. His serum creatinine continued to rise, peaking at 3.75 mg/dL, two days after admission (Figure 2). With falling urine output, worsening edema, and dyspnea (volume overload) despite intravenous Furosemide continuous infusion at 20 mg/h, renal replacement therapy (RRT) was initiated just two days after admission [12]. He subsequently was discharged and he continued outpatient in-center maintenance hemodialysis for over two and half years for ESRD. He passed away in November 2012, following an observed cardiac arrest. This picture of precipitate acute, unanticipated yet irreversible AKI resulting in ESRD needing permanent RRT is the SORO-ESRD, a newly described syndrome that we first reported in 2010 [13,15,16]. The cause of renal failure was pneumonia/septicemia on diabetic hypertensive CKD.

##### c. The Syndrome of Rapid Onset ESRD (SORO-ESRD) Following AKI on CKD in a Kidney Transplant Recipient

**Figure 3:**
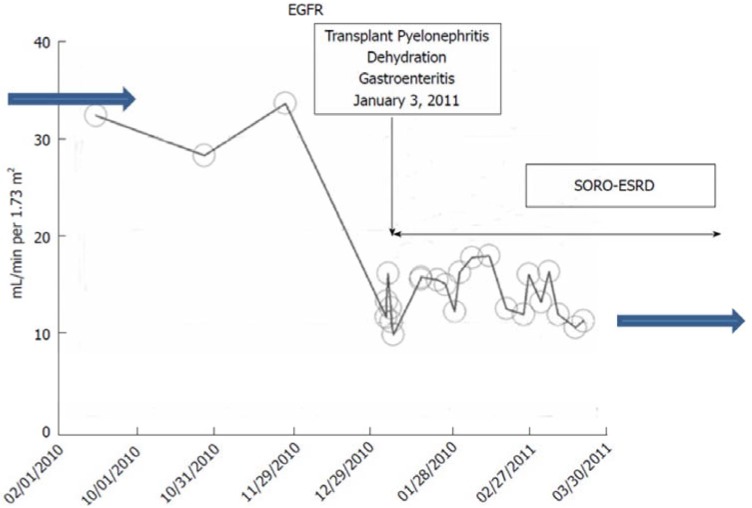
eGFR trajectory in the renal transplant recipient with a pancreas allograft following AKI-on-CKD from pyelonephritis and dehydration precipitating rapid onset yet irreversible ESRD.

A 53-year-old Caucasian female patient, with type 1 diabetes mellitus since childhood, and hypertension for over 10 years, had a simultaneous pancreas-kidney transplantation (SPK) in 2000 while on maintenance hemodialysis for ESRD. Her maintenance immunosuppression consisted of Tacrolimus, Mycophenolate Mofetil, and Prednisone. Dipstick urinalysis showed 2–3+ proteinuria, 24-h urine protein excretion in 2010 was 1.458 gm, A1c was controlled at 4.5%–6%, and an allograft renal ultrasound was normal. The patient had bilateral severe diabetic retinopathy for many years and was legally blind in the left eye. Throughout 2010, 10 years after her SPK operation, her baseline serum creatinine was 1.6–1.8 mg/dL, with an estimated glomerular filtration rate (eGFR) of ~34 mL/min/1.73 sq. m BSA, consistent with a stable renal allograft stage III CKD [12]. In January 2011, she suffered from acute allograft pyelonephritis (Escherichia Coli) that was further complicated by dehydration as a result of persistent nausea, vomiting, and diarrhea [15,16,17]. Renal allograft AKI on CKD supervened with worsening oliguria and her serum creatinine quickly increased within days to 5.16 mg/dL (Figure 3). Emergent RRT was initiated for progressive oliguric AKI, anorexia, and volume overload as hemodialysis via a tunneled central dialysis catheter on 8 January, 2011. She was subsequently referred to Mayo Clinic, Rochester, for continued care. A renal allograft biopsy at Mayo Clinic, Rochester, revealed acute tubular necrosis and chronic transplant glomerulopathy but no rejection [17]. She continued maintenance outpatient in-center hemodialysis for ESRD three times a week until January 2012, exactly one year later, when she received a second living-related renal allograft from her then-32-year-old son, again at Mayo Clinic, Rochester. Remarkably, her pancreas allograft function, part of the SPK from 2000, has nevertheless remained perfect. Her current baseline serum creatinine in February 2015 is 0.89 mg/dL, eGFR >60 mL/min/1.73 sq. m BSA. Again, this picture of precipitate acute, unanticipated yet irreversible AKI resulting in ESRD needing permanent RRT is the SORO-ESRD [13,15,16,17]. The cause of renal failure was renal allograft pyelonephritis on diabetic CKD. Notably, the renal allograft biopsy showed acute tubular necrosis.

#### ii. The NKF KDOQI CKD Staging Paradigm revisited: CKD Prediction is an Inexact Science: the Novel Concept of CKD “Progressors” and CKD “Nonprogessors”.

In 2002, the National Kidney Foundation Kidney Disease Outcomes Quality Initiative (NKF KDOQI) expert committee instituted new guidelines that established a novel chronic kidney disease (CKD) staging paradigm [18,19]. Using ranges of pre-specified eGFR, CKD was characterized into five stages in this model: CKD stage I, CKD stage II, CKD stage III, CKD stage IV, and CKD stage V [18,19]. Clearly, the assumption is that serum creatinine and eGFR trajectories in CKD patients generally follow a linear, predictable, smoothly progressive, and time-dependent curve to advance through the increasing CKD stages I through V before inexorably reaching ESRD and the need for RRT [18,19,20,21]. On the other hand, it must be acknowledged that such mathematical predictability of renal functional CKD marching through these incremental projected CKD stages and finally, inexorably ending up in symptomatic ESRD and the need for RRT is unproven, untested, and potentially flawed [13,21,22,23,24,25,26,27,28,29,30]. We recently completed an exhaustive reexamination of these phenomena regarding CKD behavior and proposed the nomenclature of CKD “progressors” and CKD “nonprogressors” to characterize the true behavior of CKD patients seen in clinical nephrology practice [21].

The following case report demonstrates the ability of CKD patients with diabetic nephropathy to remain at a later stage CKD, over long periods of time, sometimes for several years, without any further progression of CKD stages, even in older >65-year-old patients [21]. Such stability of CKD was demonstrated in a Canadian study previously [26]. These patients represent the so-called CKD “nonprogressors” [21].

### 2.2. Stable CKD V over Eight Years in a Now-79-Year-Old Caucasian Hypertensive Diabetic Male

**Figure 4:**
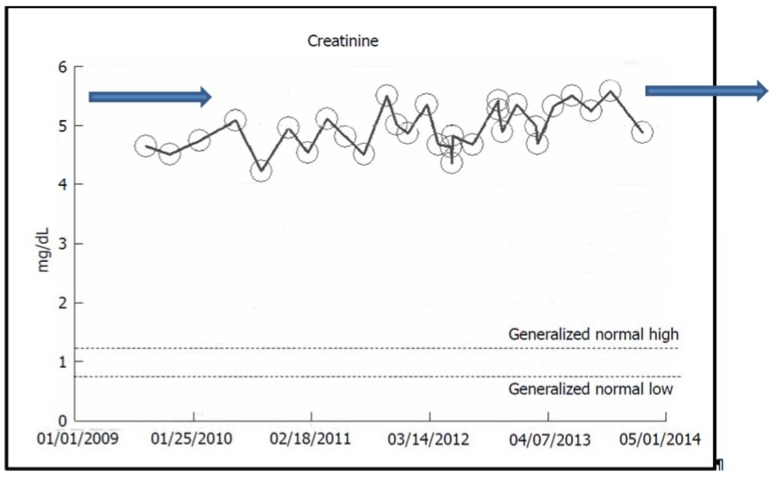
Serum creatinine trajectory in a now-79-year-old Caucasian hypertensive, diabetic male with stable CKD V between 2006 and 2014, serum creatinine of 4.5–5.5 mg/dL, eGFR 8–11 mL/min per 1.73 m^2^ BSA.

A now-79-year-old obese Caucasian man with type II diabetes mellitus and hypertension since 2001 had, over the last eight years (2006–2014), maintained a fairly stable baseline serum creatinine of 4.5–5.5 mg/dL, eGFR 8–11 mL/min per 1.73 m2 BSA, consistent with asymptomatic, otherwise stable stage V CKD (Figure 4) [21]. His A1c ranged from 7% to 9%, 24-h urine protein excretion in November 2007 was 0.74 gm with UACR ranging from 23 to 58 mg/gm (0–30). A renal ultrasound in 2005 had revealed kidneys that appeared normal. An ophthalmologic evaluation in February 2006 demonstrated the absence of changes of diabetic retinopathy. For the past four years, he has received alternate monthly courses of prophylactic short-duration courses of oral Levofloxacin treatment for recurrent UTI prophylaxis. Thus far, in February 2015, he has remained otherwise asymptomatic, still at stage V CKD, with a serum creatinine of 5.85 mg/dL, eGFR 9.4 mL/min per 1.73 m2 BSA, and has not needed RRT. The suspected cause of renal disease is normoalbuminemic diabetic hypertensive CKD.

#### iii. Predictable Pattern of CKD to ESRD Progression or the So-Called “Classic” Pattern of CKD to ESRD Progression

In a previous section on AKI-on-CKD, we had noted the features of a recently described syndrome of rapid onset ESRD (SORO-ESRD) in patients with diabetic nephropathy. We shall here describe two patients with diabetic nephropathy with features consistent with the so-called “classic” pattern of CKD to ESRD progression, where the CKD patient exhibits progressively increasing serum creatinine over years to decades before the development of symptomatic ESRD and the need for RRT. This is the predictable, linear, progressive, relentless, time-dependent, and knowable decline in renal function, with predictably increasing serum creatinine or falling eGFR, leading inexorably to ESRD and the need for RRT [18,19,20,21,31,32].

### 2.3. “Classic” Pattern of CKD-ESRD Progression in a Caucasian Hypertensive Diabetic Male

In November 2014, this patient is a 56-year-old Caucasian hypertensive, type II diabetic, obese male. He has had diabetes mellitus since age 25, with known diabetic retinopathy and multiple related procedures including vitrectomy in 2008 for vitreous hemorrhage. A1c ranged between 8.2% and 13.1% in 2007, but had improved with insulin treatment to 7%–8% from 2008 to 2015. Dipstick urinalysis showed 1–3+ proteinuria, UACR was 144 mg/gm (0–30) in March 2007, with 24-h urine protein excretion of 0.809 gm in August 2008. Nevertheless, a renal ultrasound in April 2008 was normal.

**Figure 5:**
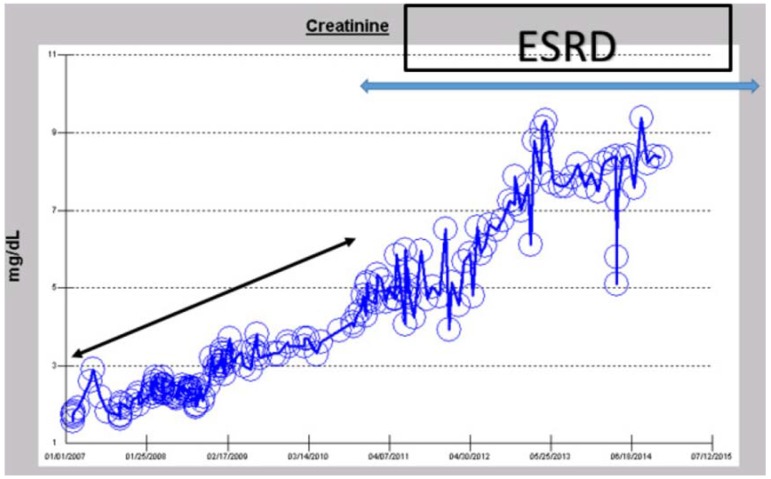
Serum creatinine trajectory in a now-56-year-old Caucasian hypertensive, diabetic male who developed predictable linear and progressive time-dependent CKD to ESRD, between 2007 and 2010, and has remained on maintenance hemodialysis from 2010–2014.

In February 2007, at age 49, he had presented with a baseline serum creatinine of 1.81 mg/dL, eGFR 43 mL/min/1.73 sq. m BSA to his primary care physician for medical follow up. Subsequently, his serum creatinine had progressively and predictably increased in a linear time-dependent manner to reach 4.72 mg/dL, eGFR 14 mL/min/1.73 sq. m BSA, by December 2010, at the age of 52, when he developed fetaures of uremia and needed the initiation of RRT for symptomatic ESRD (Figure 5). RRT in the form of hemodialysis was started via a right-tunneled internal jugular vein hemodialysis catheter. He has since then continued on in-center maintenance hemodialysis, three times weekly. His current serum creatinine in November 2014, the time of this review, after nearly four years on hemodialysis, is 8.36 mg/dL (Figure 5). The suspected cause of renal disease is diabetic hypertensive nephropathy.

#### iv. The Syndrome of Rapid Onset ESRD in a Diabetic Following AKI Complicating an Elective Cardiothoracic Procedure

**Figure 6:**
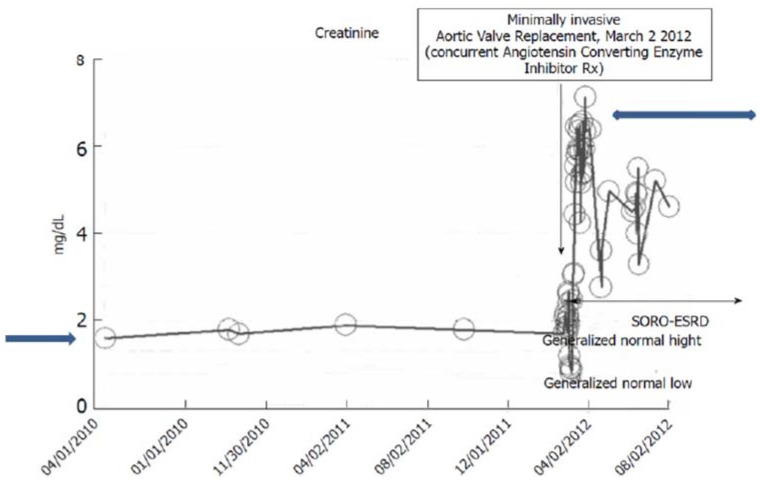
Serum creatinine trajectory, 2007–2014, with AKI in March 2012 in a 73-year-old morbidly obese, diabetic, hypertensive, CKD III Caucasian male following minimally invasive AVR for symptomatic aortic stenosis.

**Figure 7:**
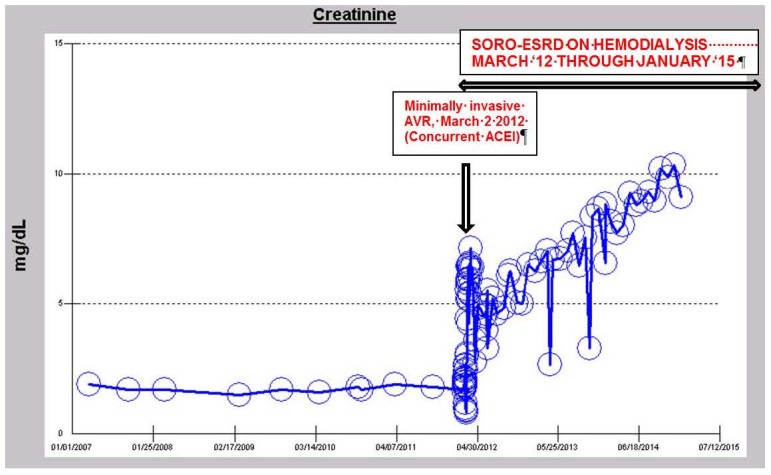
Serum creatinine trajectory, 2007–2014, with AKI in March 2012 in a 73-year-old morbidly obese, diabetic, hypertensive, CKD III Caucasian male following minimally invasive AVR for symptomatic aortic stenosis.

Between 2010 and 2012, a 73-year-old obese, hypertensive, type II diabetic male patient had maintained a stable III CKD stage diagnosis with a baseline serum creatinine of approximately 1.7 mg/dL, eGFR 43 mL/min/1.73 sq. m BSA, while continuing on concurrent ACE inhibition with Lisinopril 40 mg daily. He has had diabetes for 36 years, and is known to have diabetic retinopathy changes in both eyes including a history of left vitreous hemorrhage. A1c was 7% in 2012 with 24-h urine protein excretion of <71 mg in February 2012. A renal ultrasound in February 2012 was normal and unremarkable. In February 2012, he was admitted to the coronary care unit (CCU) with acutely decompensating heart failure [21]. The diagnosis of acute coronary syndrome was ruled out by investigation and he was found to have severe symptomatic aortic stenosis. He soon underwent minimally invasive aortic valve replacement for symptomatic aortic stenosis with a 25 mm St. Jude’s Epic stented tissue valve on 2 March, 2012. He developed acutely worsening post-operative AKI on CKD and needed hemodialysis treatment on the first post-operative day due to worsening oliguria and associated volume overload (Figure 6). His kidney function progressively worsened and he never recovered any kidney function. He has since continued outpatient in-center maintenance with hemodialysis for ESRD, three times weekly, over two and half years later. As of November 2014, the time of this review, his serum creatinine is 9.88 mg/dL (Figure 6 and Figure 7). This presentation is again consistent with the syndrome of rapid onset end stage renal disease following an elective cardiothoracic surgical operation (SORO-ESRD) [13,15,16,17]. The suspected cause of renal disease is diabetic hypertensive nephropathy with AKI aggravation following cardiothoracic procedure.

#### v. Late Onset End Stage Renal Failure from Angiotensin Blockade (LORFFAB)

We had described the syndrome of late onset renal failure from angiotensin blockade (LORFFAB) for the first time in 2005 [33,34,35]. This is the accelerated but potentially reversible iatrogenic renal failure from concurrent angiotensin blockade, occurring more often in older (>65 years old) and later-stage CKD patients, despite normal renal arteries, with the absence of traditionally acknowledged precipitating risk factors for AKI, and while remaining on the same concurrent dose of angiotensin blockade in the preceding three months or longer [33,34,35,36].

We will describe a classic case of LORFFAB in this section.

**Figure 8:**
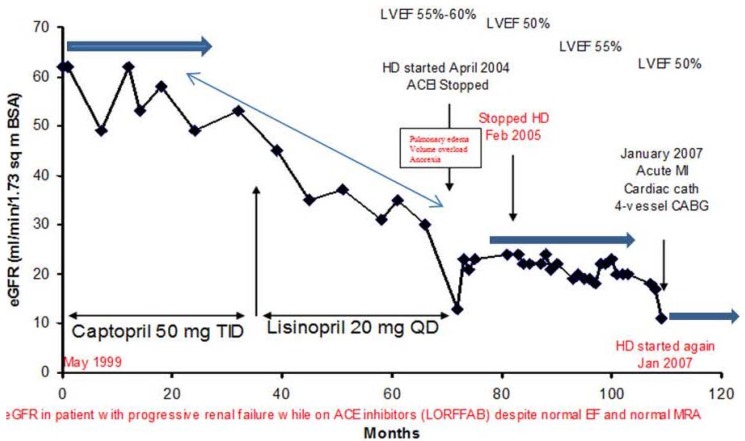
eGFR trajectory in the diabetic, hypertensive Caucasian male patient with features of LORFFAB.

**Figure 9:**
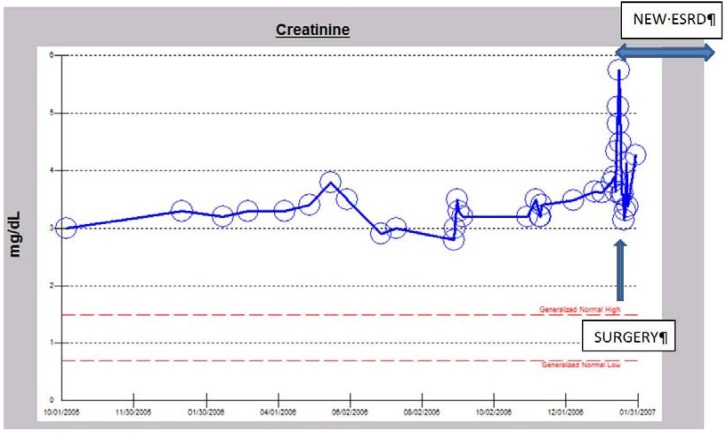
Serum creatinine trajectory in the diabetic, hypertensive Caucasian male patient with features of LORFFAB following initial recovery of renal function and hemodialysis independence before the second post-operative hemodialysis-requiring AKI-ESRD in January 2007.

A 77-year-old diabetic, hypertensive, obese Caucasian male patient presented to our Renal Unit in northwestern Wisconsin in April 2004 with symptomatic oliguric renal failure, admission serum creatinine of 4.6 mg/dL, eGFR of 13 mL/min/1.73 sq. m BSA, associated with worsening symptomatic dyspnea and volume overload (Figure 8) [34,35,36]. He had approximately a 15-year history of type II diabetes and hypertension at the time of this presentation. A1c was 6%–6.5%, UACR was 710 mg/gm (0–30), and a renal ultrasound in January 2007 showed only a mildly thinned renal cortex with both kidneys measuring 12.4 cm and 12.6 cm in length, respectively. An ophthalmology examination in May 2003 showed bilateral cataracts but no diabetic retinopathy. Emergent hemodialysis was indicated and was carried out with ultrafiltration via a temporary dialysis catheter. Lisinopril, 20 mg daily, which he had been on for the previous 21 months was promptly discontinued [34,35,36]. Amlodipine, 5 mg BID, was substituted for his antihypertensive therapy. He remained on maintenance hemodialysis for 10.5 months. Gradually, his serum creatinine improved over time while still on maintenance hemodialysis with increasing urine output (Figure 8). His serum creatinine then stabilized at about 3 mg/dL as of February 2005, and therefore hemodialysis was discontinued (Figure 9). He became hemodialysis-independent for almost two years (Figure 9). Unfortunately, in January 2007, at the age of 79, still off hemodialysis, with otherwise stable CKD IV, the patient experienced a heart attack, followed by a diagnostic cardiac catheterization, and subsequently underwent a four-vessel coronary artery bypass graft surgical operation in our hospital. Successively, he again experienced worsening post-operative AKI requiring, once again, the initiation of hemodialysis, post-operatively (Figure 9). He was however transferred out from our hemodialysis unit to an out-of-state cardiac rehabilitation center in Minneapolis, Minnesota, and he was lost to follow up [36]. The suspected cause of renal disease is diabetic hypertensive nephropathy with AKI superimposed.

#### vi. Peri-Operative AKI in Diabetic Nephropathy and the Association with Intra-Operative Hypotension While Concurrently on Angiotensin Inhibition

The role of concurrent angiotensin inhibition alone, without the involvement of the combination “triple whammy” medications in exacerbating post-operative AKI, continues to generate significant controversy in the nephrology literature [31,36,37,38,39]. The duration of diabetes mellitus was 10 or more years, A1c was 6.9% in May 2014, UACR was normal previously, but was elevated to 628 mg/gm (0–30) on presentation with AKI. The renal ultrasound was normal. There were no ophthalmology records for review. However, it would appear that on the balance, accruing evidence favors the notion that more so with prevalent intra-operative hypotension, concurrent angiotensin inhibition results in higher rates of post-operative AKI when compared to situations with its pre-emptive withdrawal pre-operatively [31,36,37,38,39]. The later approach of a pre-operative, pre-emptive temporary withdrawal of angiotensin inhibition especially in the older (>65-year-old and later stage CKD) patients is indeed one of the cornerstones of our newly introduced concept of “renoprevention” [21,40]. We now report a case of post-operative AKI apparently exacerbated by concurrent angiotensin inhibition here.

**Figure 10:**
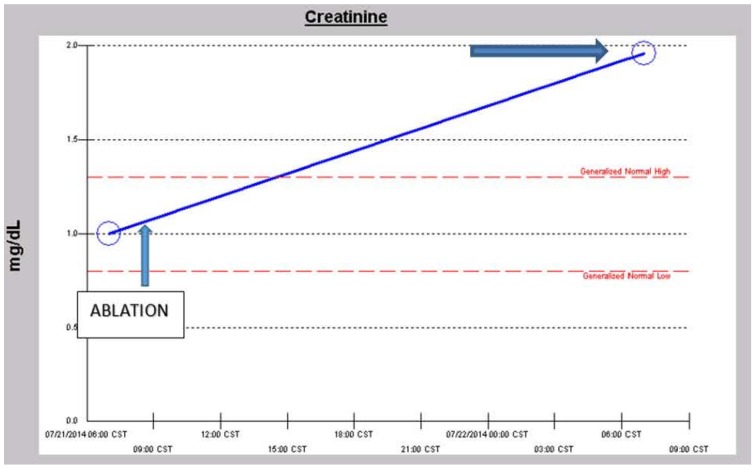
Serum creatinine trajectory on first post-operative day after elective ablation procedure for symptomatic atrial fibrillation.

**Figure 11:**
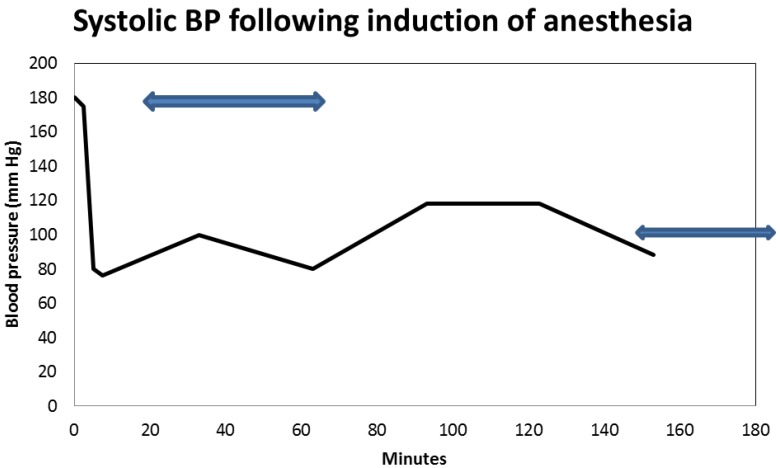
Intra-operative systolic blood pressure translations after induction of anesthesia during elective ablation procedure for symptomatic atrial fibrillation.

**Figure 12:**
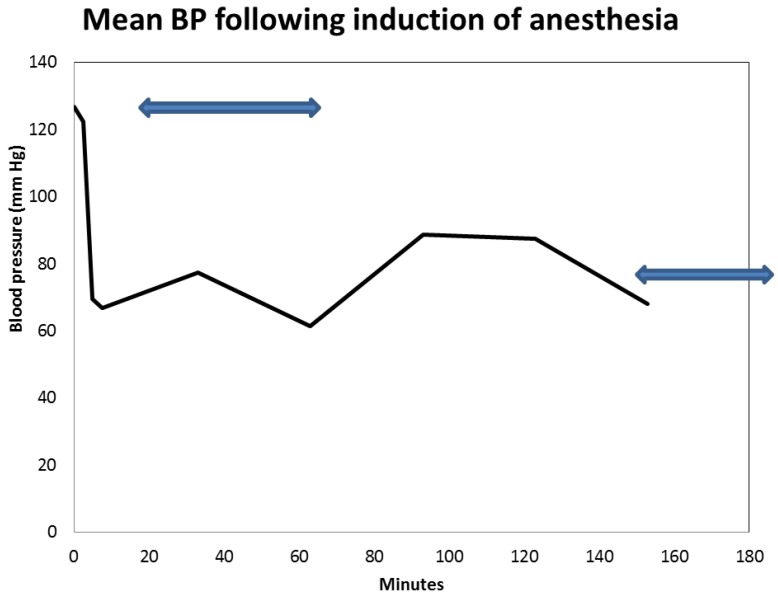
Intra-operative mean blood pressure translations after induction of anesthesia during elective ablation procedure for symptomatic atrial fibrillation.

**Figure 13:**
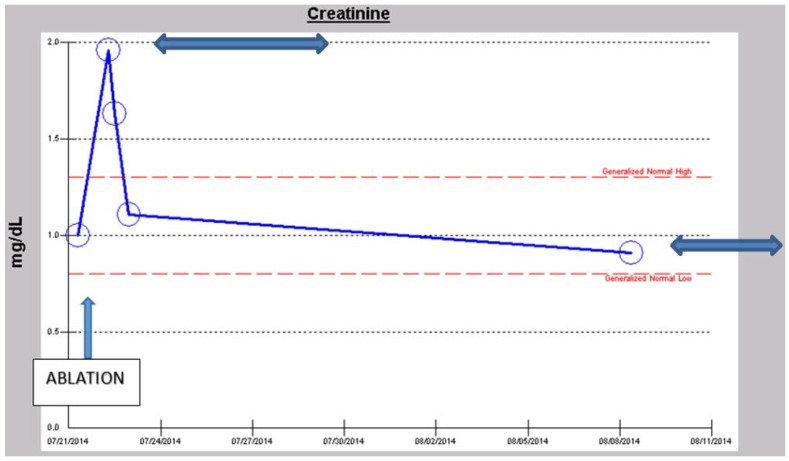
Serum creatinine trajectory two weeks after elective ablation procedure for symptomatic atrial fibrillation demonstrating complete recovery from the AKI.

A 57-year-old obese, hypertensive, type II diabetic Caucasian male patient underwent an elective pulmonary vein isolation and ablation procedure for symptomatic atrial fibrillation in July 2014. Concurrent outpatient oral medications included Chlorthalidone, Metformin 1 g BID, and Lisinopril 40 mg daily. Post-operative AKI triggered a nephrology consultation. Baseline serum creatinine was 1.0 mg/dL, GFR 81 mL/min/1.73 sq. m BSA, CKD stage II. On post-operative day one, serum creatinine had increased to 1.96 mg/dL (Figure 10). At this time, the patient was normotensive and all medical floor BP recordings before and after the procedure were noted to be normal. We had been informed that the procedure “went well without complications.” However, an urgent review of the intra-operative anesthesia records revealed significant hypotension during the over-four-hour surgical procedure (Figure 11 and Figure 12). He was nonoliguric and otherwise asymptomatic except for mild lightheadedness. The patient was therefore managed conservatively. Lisinopril and Metformin were promptly discontinued. Kidney function subsequently quickly improved. At discharge, he was placed back on Lisinopril with stable kidney function thereafter. His latest serum creatinine later in August 2014, the following month, was 0.91 mg/dL, eGFR of 91 mL/min/1.73 sq. m BSA (Figure 13).

First and foremost, his admission blood pressure was 156/110, uncontrolled hypertension. In the operating room, his pre-operative systolic blood pressure was 180 mm Hg and this quickly dropped to as low as 80 mm Hg within 10 minutes of the induction of anesthesia (Figure 11). It is our hypothesis that if his ACE inhibition was pre-emptively withheld three to five days before the planned elective ablation procedure (renoprevention), and that if his intra-operative systolic blood pressures were maintained at a higher level of 90–100 mm Hg, the post-operative AKI observed would have not occurred [40,41]. This would have meant reduced patient morbidity, reduced length of stay and major savings in U.S. healthcare dollars [41]. The suspected cause of renal disease is reversible AKI, likely from acute tubular necrosis from prolonged hypotension in a predisposed patient.

## 3. Conclusions

### 3.1. A Need for More Preventative Renal Medicine–Renoprevention Revisited: The Introduction of the New Innovative CKD Express © IT Software Application

In this review of serum creatinine trajectories in kidney disease, we have examined and reported on the multifarious behavior of serum creatinine in AKI; in CKD to ESRD progression, including SORO-ESRD; in otherwise non-progressing CKD; in LORFFAB; and in post-operative AKI associated with iatrogenic intra-operative hypotension on concurrent ACE inhibition. It must be acknowledged here that the monitoring of trajectories of changes of kidney function using either serum creatinine or eGFR yields similar diagnostic and prognostication data and results. Clearly, there is a broad spectrum of renal outcomes following AKI. Moreover, CKD in all its stages can be “progressors” *vs.* “nonprogressors”, or “nonimprovers” *vs.* “improvers” [21,26,42,43,44,45,46]. From the foregoing, clearly, CKD prediction and prognostication is an inexact science [21,26,42,43,44,45,46]. The alternate side of CKD unpredictability is when patients have remained in a later-stage CKD for years with no evidence of progression [21]. CKD care must therefore be individualized in both diabetic and non-diabetic CKD patients. We recently conceived new IT software, The CKD Express © IT Software, currently in development [30,47,48,49]. This software, is an adware that can complement EMR systems and would automatically collate, analyze, and track serum creatinine and/or eGFR trajectories of all CKD patients in its database; make diagnostic and prognostic decisions using artificial intelligence (AI) and decision support system (DSS) tools; and would advise the supervising mid-level provider on the next right step to take in the CKD patient management paradigm of care [30,47,48,49]. The incorporation of this software application into existing electronic medical records (EMR) systems would go a long way in bridging this yawning gap in our knowledge and understanding of CKD initiation, propagation, and progression. Furthermore, its introduction into general medicine and practice around the world could potentially save billions of scarce health care dollars in pre-dialysis chronic kidney disease care [49].

### 3.2. GFR Loss in Diabetic Patients without or Independent of Albuminuria

As was evident in our case series, not uncommonly, diabetic patients show evidence of renal disease as measured by serum creatinine and/or eGFR changes, despite the presence of significant albuminuria [2,3,4,5,6,50,51,52,53]. In a 2006 observational study, Middleton *et al.* examined 7596 patients with diabetes in primary and secondary care in Salford, United Kingdom, and demonstrated that the combination of abnormal creatinine and albuminuria had an improved performance but still failed to detect a large number of patients with CKD (sensitivity 82.4%, specificity 75.4%) [50]. In a related study published in 2007, a survey of 5072 patients with diabetes in 17 practices in Surrey, Kent, and Greater Manchester, in the United Kingdom, recruited between 2003 and 2004, revealed that 31% had clinically significant CKD (defined as eGFR <60 mL/min per 1.73 m (2)) [51]. Of patients with diabetes with eGFR <60 mL/min per 1.73 m (2), 63% had normoalbuminuria [51]. Similarly, in a cross-sectional Japanese study published in 2010, Ito *et al.* analyzed 338 patients with type 2 diabetes mellitus and showed clear dissociations between intima-media thickness and CKD stages with the various degrees of albuminuria and normoalbuminuria [52]. Halimi, in a recent review, concluded that progressive deterioration of renal function due to diabetes without developing significant proteinuria is common, is seen fairly frequently, and can affect 50% of patients with renal insufficiency [53].

Finally, we will conclude that the study of individual patient-level serum creatinine trajectories, albeit a neglected and forgotten diagnostic methodology for diabetic CKD prognostication and prediction, is a most useful diagnostic tool, both in the short-term and in the long-term practice of nephrology in particular, and medicine, in general [14]. The analysis of serum creatinine trajectories, both in real-time and retrospectively, does indeed provide supplementary superior diagnostic and prognostic insights in the management of the nephrology patient [14].
